# Right Heart Transvalvular Embolus with High Risk Pulmonary Embolism in a Recently Hospitalized Patient: A Case Report of a Therapeutic Challenge

**DOI:** 10.1155/2015/481357

**Published:** 2015-08-31

**Authors:** Gyanendra Kumar Acharya, Ajibola Monsur Adedayo, Hejmadi Prabhu, Derek R. Brinster, Parvez Mir

**Affiliations:** ^1^Department of Internal Medicine, Wyckoff Heights Medical Center, Brooklyn, NY 11237, USA; ^2^Division of Cardiology, Department of Internal Medicine, Wyckoff Heights Medical Center, Brooklyn, NY 11237, USA; ^3^Department of Cardiothoracic Surgery, Lenox Hill Hospital, North Shore-Long Island Jewish Health System, New York, NY 10075, USA; ^4^Division of Pulmonary and Critical Care Medicine, Department of Internal Medicine, Wyckoff Heights Medical Center, Brooklyn, NY 11237, USA

## Abstract

Thrombus-in-transit is not uncommon in pulmonary embolism but Right Heart Transvalvular Embolus (RHTVE) complicating this is rare. A 54-year-old obese male with recent hospitalization presented with severe dyspnea and collapse. Initial investigations revealed elevated d-dimer and troponin. CTA showed saddle pulmonary embolus and bedside echocardiogram revealed right ventricular (RV) pressure overload and dilatation (RV > 41 mm), McConnell's sign, and mobile echodensity attached to tricuspid valve. Patient was immediately resuscitated and promptly transferred for surgical embolectomy under cardiopulmonary bypass. A long segment of embolus traversing through the tricuspid valve and extensive bilateral pulmonary artery embolus were removed. IVC filter was placed for a persistent right lower extremity DVT. Hypercoagulable work-up was negative. Patient continued to do well after discharge on Coumadin. Open embolectomy offers great promises where there is no consensus in optimal management approach in such patients. Bedside echocardiogram is vital in risk stratification and deciding choice of advanced PE treatment.

## 1. Introduction

Right-heart-thrombus is not uncommon in acute massive pulmonary embolism (PE) and appears to increase mortality substantially [1, 2]. It requires emergency treatment, but there is no consensus regarding optimal management. Our patient presented with massive PE along with a unique echocardiographic finding of a long Right Heart Transvalvular Embolus (RHTVE) traversing the tricuspid valve. In appropriate settings, such patients would benefit from emergency surgical embolectomy.

## 2. Case Presentation

**Figure 1:**
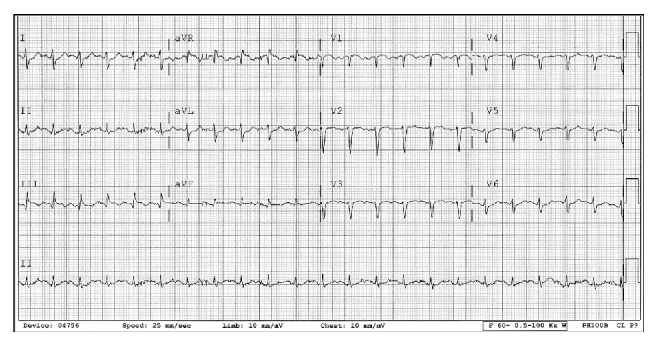
12-lead EKG showing right ventricular strain pattern.

**Figure 2:**
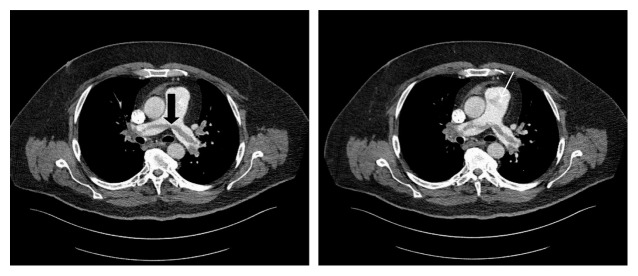
Computed Tomographic Pulmonary Angiogram (CTPA) showing massive saddle pulmonary embolus (bold arrow) with hypodense area at the root of pulmonary artery consistent with other emboli (white arrow).

**Figure 3:**
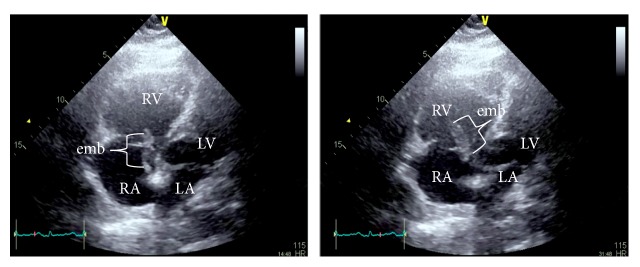
Transthoracic echocardiogram, apical 4-chamber view, showing RV pressure overload with enlarged RV diameter (>41 mm) and a long echodensity (embolus) attached to TV extending in both RA and RV (RHTVE); LA: left atrium; RV: right ventricle; RA: right atrium; LV: left ventricle; TV: tricuspid valve; emb: embolus; RHTVE: Right Heart Transvalvular Embolus.

54-year-old obese male presented to ER with episodic dyspnea and collapse without loss of consciousness associated with palpitation, chest discomfort, and diaphoresis. He was recently hospitalized for colonic diverticular abscess that was drained. Medical history includes hypertension, CAD, and asthma. He denied smoking/illicit drugs. Examination was remarkable for tachycardia, hypotension, and mild hypoxia. Laboratory findings revealed elevated lactic acid, troponin, d-dimer, and abnormal EKG (Table 1, Figure 1). Urgent CT angiogram showed extensive bilateral pulmonary embolus (Figure 2), and bedside echocardiogram revealed right ventricular (RV) pressure overload with enlarged RV (>41 mm) and mobile echodensity attached to tricuspid valve that appeared to extend into right atrium and ventricle, RHTVE (Figure 3).

**Figure 4:**
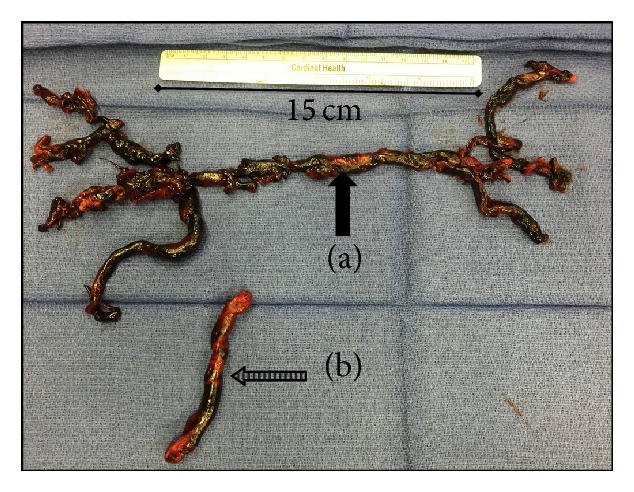
Postoperative demonstration of (a) a large (~25 cm) pulmonary embolus* en bloc* (bold arrow) retrieved from the right and left pulmonary arteries; (b) a long segment of embolus (doted arrow) traversing along tricuspid valve (TV). Both of them were removed in the same open embolectomy under cardiopulmonary bypass procedure.

Patient was resuscitated with intravenous fluid, 40% Ventimask, and anticoagulated with intravenous heparin. After interdisciplinary discussion, patient was transferred to the nearest tertiary facility for open embolectomy with cardiopulmonary bypass. On follow-up with the tertiary center, a long segment of blood clot traversing the right atrium and ventricle (Figure 4(b)) and extensive bilateral pulmonary artery embolus (Figure 4(a)) were removed. Additionally, IVC filter was placed for a continued right lower extremity thrombus extending from common femoral to the posterior tibial vein. Work-up for hypercoagulable studies was negative. He was discharged on Coumadin and continues to do well on follow-up with us.

## 3. Discussion

Thrombus-in-transit is not an unusual presentation in high risk PE [3]. This case chronicles a rare large embolus traversing through tricuspid valve from right atrium to right ventricle and we termed this as Right Heart Transvalvular Embolus (RHTVE). High risk PE may be massive or submassive. It often results from embolization of proximal deep vein thrombus in the lower extremities. Massive PE is characterized by sustained hypotension with SBP <90 for at least 15 minutes or requiring intravenous vasopressor(s). It may also be a state of bradycardia with heart rate <40 associated with signs and symptoms of shock or pulselessness. Submassive PE is that without hypotension associated with RV dysfunction (right bundle branch block/right heart strain, McConnell's sign, or elevated BNP) or evidence of myocardial injury (troponin >0.1) [4].

Pulmonary artery pressure increases abruptly once the embolus occludes > 30–50% of the total cross-sectional area of pulmonary arterial bed. This anatomical obstruction and reactive vasoconstriction induced by release of thromboxane A2 and serotonin together increase pulmonary vascular resistance [5]. The RV adapts via frank-sterling mechanism to maintain BP by generating counterpressure (persistent contraction) against rising pulmonary artery pressure, this adaptation is limited, and soon RV dilatation ensues precipitating hemodynamic instability [5]. Secondly, the resulting ventilation perfusion mismatch and decreased cardiac output contribute to hypotension, hypoxemia, and eventual respiratory failure [5]. Hypoxemia and increased myocardial demand may explain the lactic acidemia and elevated troponin in our patient.

Investigators have associated massive and submassive PE with 52.4% and 14.7% 90-day mortality, respectively [6]. One study concluded that hemodynamically unstable PE is associated with >25% in-hospital mortality [7]. Echocardiographic and CTPA findings of RV dysfunction were shown as independent predictors of 30-day mortality while elevated troponin increases short-term risk of death up to fivefold [8–10]. Resuscitative measures, with judicious intravenous fluid administration to stabilize SBP and 40% Ventimask to decrease work of breathing, provided the critical window for urgent CTA and echocardiogram. Echocardiogram served as a cornerstone for both diagnosis and risk stratification (identified RHTVE).

Currently, there is no randomized trial that supports or contravenes open embolectomy; it remains a vital treatment option in advanced management of PE. Open embolectomy has its attendant risks and complications but early surgical referral can skew outcome favorably [11]. Investigators have suggested open embolectomy as a reasonable option provided that patients are identified before onset of cardiac arrest [11–14]. Our patient presented with massive PE and a unique Echo finding of RHTVE. After interdisciplinary consultations, we concluded that surgical option would not only recover the thrombus in right heart and central branches of pulmonary artery before further deterioration, but also avert the possibilities of failed thrombolysis. We opted for invasive approach (open embolectomy with cardiopulmonary bypass) for the following reasons. Bedside 2D echocardiogram revealed a long embolus traversing through the tricuspid valve on the background of massive saddle embolus extending in main branches of pulmonary artery. The right ventricle was already on massive pressure overload with dilatation and shifting of ventricular septum towards left ventricle. Further clot burden to pulmonary artery (by imminent dislodging the transvalvular clot) would have been catastrophic in the patient. So it was decided to immediately transfer the patient to operating room for open embolectomy under cardiopulmonary bypass procedure. Clot propagation was still ongoing as evidenced by presence of large thrombus in the femoral and tibial veins (IVC filter was placed). This could continue to be deposited distally. Catheter-directed thrombolysis may be complicated by deposition of fragments in terminal branches of the pulmonary arteries, which may result in chronic pulmonary embolism/hypertension. Other treatment approaches may still be complicated with cardiac arrest with even higher mortality. The benefits outweighed the risk. Open embolectomy offers great promises in selected patients who are relatively stable (absence of cardiac arrest) where there is no consensus yet in optimal management approach.

In conclusion, bedside echocardiogram is vital in risk stratification and deciding choice of advanced PE treatment. High risk PE patients with RHTVE can benefit from emergent open embolectomy with cardiopulmonary bypass.

## Supplementary Material

This is a 2D-Echocardiogram video clip, apical 4-chamber view, of the heart showing effect of RHTVE/saddle embolus. The RV is seen markedly enlarged with the visualized interventricular septum “outpouching” towards the LV presumably under extreme RV pressure overload (secondary to saddle embolus in the pulmonary arteries; not shown here). Also visible is the embolus (RHTVE) traversing the tricuspid valve with greater portion in the RV and a proximal end in the RA. See also case presentation for more explanation. RHTVE- Right Heart Transvalvular Embolus; RV- Right Ventricle; LV- Left Ventricle.

## Figures and Tables

**Table 1:** Baseline laboratory findings in the patient at presentation.

SN	Tests	Values	Reference value
(1)	CBC		
	WBC	12.60	4.50–10.9 K/mm^3^
	RBC	4.92	3.8–5.2 × 10^6^/mm^3^
	Hb	14.2	12.2–15 gm/dL
	Platelets	212.0	130–400 × 10^3^/mm^3^
	ESR	30.0	0–35 mm/hr

(2)	Chemistry		
	Sodium	137.0	136–145 mmol/L
	Potassium	4.9	3.5–5.1 mmol/L
	BUN	16.0	6–20 mg/dL
	Creatinine	1.0	0.6–1.1 mg/dL
	LDH	482.0	100–190 IU/L
	Troponin I	0.122	0–0.1 ng/ml
	Lactic acid	5.0	0.5–2.2 mmol/L

(3)	Basic coagulation profile		
	PT	13.8 sec	9.70–13.20 sec
	INR	1.21	0.86–1.16 (ratio)
	PTT	35.3	20.30–36.00 sec
	d-dimer	7205.26	400–560 ng/mLFEU

## Abbreviations

BNP: Brain natriuretic peptide
BUN: Blood urea nitrogen
CAD: Coronary artery disease
CBC: Complete blood count
CT: Computed tomography
CTA: Computed tomographic angiogram
CTPA: Computed Tomographic Pulmonary Angiogram
Echo: Echocardiogram
EKG: Electrocardiogram
emb: Embolus
ER: Emergency room
INR: International normalized ratio
IVC: Inferior vena cava
LA: Left atrium
LDH: Lactate dehydrogenase
LV: Left ventricle
PE: Pulmonary embolism
PT: Prothrombin time
PTT: Partial thromboplastin time
RA: Right atrium
RBC: Red blood cells
RHTVE: Right Heart Transvalvular Embolus
RV: Right ventricle
SBP: Systolic blood pressure
TV: Tricuspid valve
WBC: White blood cells.
